# Does access to a colorectal cancer screening website and/or a nurse-managed telephone help line provided to patients by their family physician increase fecal occult blood test uptake?: results from a pragmatic cluster randomized controlled trial

**DOI:** 10.1186/1471-2407-14-263

**Published:** 2014-04-16

**Authors:** Kathleen Clouston, Alan Katz, Patricia J Martens, Jeff Sisler, Donna Turner, Michelle Lobchuk, Susan McClement, Gary Crow

**Affiliations:** 1 Department of Family Medicine Research, Faculty of Medicine, University of Manitoba, 208 Baisinger Drive, Winnipeg R2N 4H7 MB, Canada; 2 Departments of Family Medicine and Community Health Sciences, Faculty of Medicine, University of Manitoba, P228-770 Bannatyne Ave, Winnipeg, MB R3E 0 W3, Canada; 3 Department of Community Health Sciences, Manitoba Centre for Health Policy, Faculty of Medicine, University of Manitoba, 408-727 McDermot Ave, Winnipeg, MB R3E 3P5, Canada; 4 Primary Care Oncology Program, CancerCare Manitoba, 675 McDermot Ave, Room ON2038, Winnipeg R3E 0 V9, MB, Canada; 5 Provincial Director of Population Oncology, CancerCare Manitoba, 675 McDermot Ave, 4th Floor Executive Offices, Winnipeg R3E 0 V9, MB, Canada; 6 Faculty of Nursing, Helen Glass Centre for Nursing, University of Manitoba, Room 315-89 Curry Place, Winnipeg, MB R3T 2 N2, Canada; 7 Faculty of Nursing, University of Manitoba; CancerCare Manitoba, Office # 3017, 675 Mc Dermot Avenue, Winnipeg R3E 0 V9, MB, Canada; 8 Faculty of Agriculture, Department of Animal Science, University of Manitoba, 230 Animal Science Building, Winnipeg, MB R3T 2 N2, Canada; 9 CIHR/CCMB Primary Care Oncology Research Team, 385 Main Street, Winkler, MB R6W 1J2, Canada

**Keywords:** Colorectal cancer screening, Fecal occult blood test, Community-based family practice, Community-based primary healthcare research, Cluster randomized controlled trial, Pragmatic, Patient decision aid, Integrated knowledge translation, Knowledge exchange

## Abstract

**Background:**

Evaluation of the effectiveness of a patient decision aid (nurse-managed telephone support line and/or colorectal cancer screening website), distributed to patients by their family physician, in improving fecal occult blood test (FOBT) colorectal cancer screening rates.

**Methods:**

A pragmatic, two arm, cluster randomized controlled trial in Winnipeg, Manitoba, Canada (39 medical clinic clusters; 79 fee-for-service family physicians; 2,395 average risk patients). All physicians followed their standard clinical screening practice. Intervention group physicians provided a fridge magnet to patients that facilitated patient decision aid access. Primary endpoint was FOBT screening rate within four months.

Multi-level logistic regression to determine effect of cluster, physician, and patient level factors on patient FOBT completion rate. ICC determined.

**Results:**

Family physicians were randomized to control (n = 39) and intervention (n = 40) groups. Compared to controls (56.9%; n = 663/1165), patients receiving the intervention had a higher FOBT completion rate (66.6%; n = 805/1209; OR of 1.47; 95% confidence interval 1.06 to 2.03; p < 0.02). Patient aid utilization was low (1.1%; 13/1,221) and neither internet nor telephone access affected screening rates for the intervention group. FOBT screening rates differed among clinics and physicians (p < 0.0001). Patients whose physician promoted the FOBT were more likely to complete it (65%; n = 1140/1755) compared to those whose physician did not (51.1%; n = 242/470; p < 0.0001; OR of 1.54 and 95% CI of 1.23 to 1.92). Patients reporting they had done an FOBT in the past were more likely to complete the test (70.6%; n = 1141/1616; p < 0.0001; 95% CI 2.51 to 3.73) than those who had not (43%; n = 303/705). Patients 50–59 years old had lower screening rates compared to those over 60 (p < 0.0001). 75% of patients completing the test did so in 34 days.

**Conclusion:**

Despite minimal use of the patient aid, intervention group patients were more likely to complete the FOBT. Powerful strategies to increase colorectal cancer screening rates include a recommendation to do the test from the family physician and focusing efforts on patients age 50–59 years to ensure they complete their first FOBT.

**Trial registration:**

Trial registration number: clinicaltrials.gov identifier NCT01026753.

## Background

In Canada, colorectal cancer (CRC) is the third most common cancer diagnosis and the second most common cause of death due to cancer (12% of all cancer deaths in 2012) [1]. Prevention and early detection of CRC is possible through routine fecal occult blood test (FOBT) screening of individuals 50 to 74 years of age [2,3] as it has been estimated that if 70% of eligible Canadians completed an FOBT every two years, followed up by colonoscopy for positive FOBTs, the CRC mortality rate could be reduced by 17% [4,5]. Uptake of fecal occult blood test screening in Canada and Manitoba are sub-optimal. Various surveys have indicated rates of self-reported FOBT uptake for Manitobans of 38% to 64% [6-8]. Improvements in FOBT uptake in those 50 to 59 years of age remain warranted based on the estimation that, in 2012, the highest burden of new cancer diagnoses (28%) and death (22%) will be in Canadians between the ages 60 to 69 years of age [1] and only 27.5% of Canadians 50 to 59 years of age report being screened for CRC [9]. The two most common reasons Canadians provide for not completing an FOBT are: (1) they do not see the need for the test and as they are not experiencing any symptoms (49.3%) of colorectal cancer and (2) they were not told by their doctor to do the test. Only 38% of Manitobans report having a discussion about colorectal cancer screening with their physician [8,9]. Approximately 60% of Manitobans do not understand that screening begins before symptoms appear [9]. Seventy-two percent of patients report being up-to-date with screening after having discussed CRC screening with their doctors, whereas only 33% of those who did not have this conversation were up-to-date with CRC screening [9]. However, physicians estimate it takes approximately four minutes to do a good job of explaining CRC and relevant screening options [10] which represents 27 to 40% of the total time for the periodic (annual) health examination. The complexities of clinical practice, including caring for patients with increasingly complex multiple morbidities [11,12] and time constraints, impact on the time available to adequately address all health concerns during the periodic (annual) health examination, especially prevention and screening [13].

Higher FOBT completion rates have been reported in studies of intervention strategies utilizing one-on-one patient contact with registered nurses within the medical clinic. However, this strategy can be costly and time consuming [10]. It is also uncommon in most community-based fee-for-service clinical practices. A multimedia educational computer program was shown to be as effective as usual nurse counseling in educating patients and achieving adherence to FOBT screening [14]. In 2010, 73% of Manitobans had home internet access of which approximately 75% went online every day in a typical month. 74% percent of females searched for information about health or medical conditions compared to 66.7% of men [15]. The role of the internet in supporting positive health promotion behaviors is currently unknown. Nurse-managed telephone support for cardiovascular disease patients has been shown effective in managing the disease [16]. We postulated that a patient aid, providing colorectal cancer and screening information that was given to patients by their family physician, would increase patient FOBT completion rates. The patient aid was designed to support family physicians in their goal to increase their patient’s colorectal cancer screening rates while addressing the constraints of clinical practice. It contained the URL address and telephone number for a colorectal cancer information and screening website and nurse-managed telephone support line, both specifically designed for the study.

A systematic review of the literature has reported that many cluster randomized cancer screening intervention trials (19 CRC only and 6 CRC plus other sites) had deficiencies in the application of correct statistical procedures for the outcome analysis which led to unjustified rejection of the null hypothesis [17]. Our study design accounts for the complexities of cluster randomization at both the clinic and family physician levels and is able to contribute intraclass correlation coefficients for these outcome variables.

## Methods

Community-based fee-for-service family physicians (FP) were recruited within the city of Winnipeg, Manitoba, Canada (population 685,000) to participate in this pragmatic cluster randomized controlled trial. In order to be eligible for the study, FPs had to be in full or part-time regular solo or group family practice. Some participating physicians were involved in local primary care quality improvement initiatives (Uniting Primary Care and Oncology and Physician Integrated Network) [18,19] that could impact CRC screening rates, therefore this was addressed in our analyses. Each physician’s patients were eligible for participation in the study if they were 50 to 74 years of age, had no symptoms of CRC, no personal history of CRC, polyps or diseases of the colon requiring monitoring by colonoscopy (Crohn’s Disease or Ulcerative Colitis). Patient consent was obtained by the physician’s support staff. All of a physician’s patients received the same experimental treatment (control or intervention) based on the cluster randomization. Hence, patient consent was obtained post randomization. The study protocol was approved by the University of Manitoba Health Research Ethics Board.

Randomization of clusters was conducted by a biostatistician using a computer-generated list. Clusters were block randomized based on the number of collaborating FPs within a practice. An absolute increase in FOBT screening rates of 15% from the most current FOBT completion rate know at the time of the study (42% in Manitoba) was considered clinically significant [15]. Sample size was determined by a biostatistician using PASS (Power and Sample Size, 2002) software [19] and a computer simulated ICC value of 0.6 [20]; a very conservative estimate of the minimum detectable effect size given the proposed sample size. Based on logistic regression, a cluster size of 41 clusters each enrolling 30 to 35 patients (1230 observations) will achieve 90% power at a 0.05 significance level to detect a change from the baseline value of 0.400 to 0.550 and corresponds to an odds ratio of 1.833. In the event that family physician collaboration/retention and/or patient recruitment proved to be more difficult than anticipated, a logistic regression with a cluster size of 28 (840 observations) would have achieved 80% power with the same outcomes.

All FPs collaborating in the study followed their usual clinical practice for CRC screening with the FOBT during the patient periodic (annual) health examination. The rationale for not customizing this portion of the study was to optimize FP collaboration and to simulate “real world” clinical practice. The study coordinator (KC) enrolled family physicians/clusters and family physicians enrolled patients. The intervention was applied at the medical clinic (cluster) level; each cluster was randomized to either the control or intervention group. The randomization sequence was concealed until interventions were assigned by the study coordinator. Patients, as well as those assessing the outcomes, were blinded to treatment group designation. FPs in the intervention group provided their patients with the patient aid (refrigerator magnet) during the physician visit. This magnet provided information on how to access a study specific colorectal cancer information and screening nurse-managed telephone support line (telephone number) and website (URL). Access to the telephone support line and/or website for the intervention group was patient initiated and was tracked with the assigned study-specific, identification number.

Factors influencing FOBT completion rate were assessed at three levels: medical clinic cluster, individual family physician, and patient. Patient aid utilization was also evaluated. Data were obtained from three study specific surveys: the In-Clinic Patient Survey (completed prior to the visit by all patients of each FP), Post-Study Patient Follow-Up telephone survey (n = 10 patients per family physician in both treatment groups), and Family Physician Surveys (provided to all FPs involved in the study. Time to completion of the FOBT is a secondary outcome variable reported along with relevant family physician and patient post-study survey responses. Further details regarding the study methods can be found in the published protocol paper [21].

The primary dependent variable was the binary response of patients completing the FOBT within four months. However, patients were eligible to complete the FOBT test after the four month end-point as this was an arbitrary cut-off point based on the literature suggesting most patients complete the FOBT within four to six weeks of receiving the test [10]. This information was obtained by the study coordinator (KC) directly from each family physician. Factors affecting FOBT uptake included patient characteristics as well as those of the physician and clinic. The trial protocol [19] defines a multi-level investigation facilitating analysis of the intraclass correlation coefficient (ICC) for each primary group-level outcome (medical cluster, family physician, and patients) [22]. A number of statistical models were used to test hypotheses about factors and their interactions. Most models were mixed models with factors such as age, gender, socioeconomic status (SES), use of an electronic medical record (EMR), and practice participation in primary care reform initiatives considered as fixed effects. Clinic and physician were considered to be random effects and to be representative of the population of physicians and the clinics to which they belonged. The GLIMMIX procedure of SAS 9.2 was used for this analysis; factors and their interactions were considered significant using a type I error rate of 0.05.

## Results

**Figure 1:**
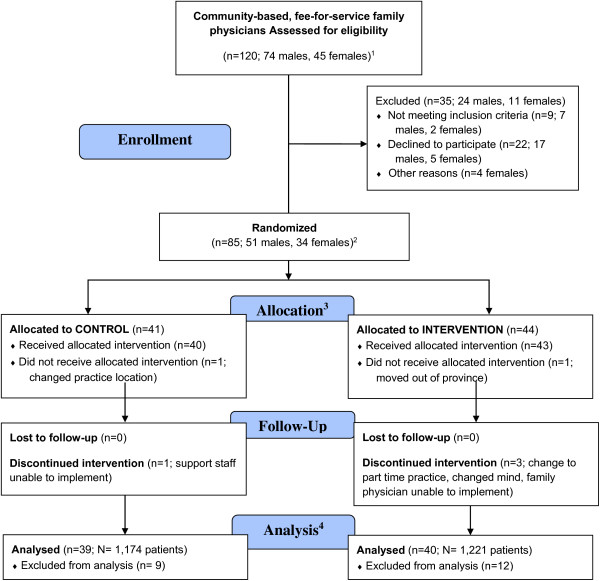
**CONSORT flow diagram for family physician recruitment**^**5**^**.**^1^Representing 30 community-based medical clinics. ^2^Representing 23 medical clinics (of which 3 contained both control and intervention clusters). Each family physician was asked to enroll 30–35 patients. Total number of patients was n = 2,395; 915 males, 1473 females. ^3^Control group represented by 13 clinics and 19 clusters; intervention group represented by 12 clinics and 20 clusters. See Table 5 for detailed description of clusters. ^4^Number of patients analyzed; control (n = 1,174) and intervention (n = 1,221). ^5^Based on the CONSORT flow diagram [23].

Community-based fee-for-service family physicians were recruited over eight months (January, 2010 to August, 2010). Study implementation occurred between September and October 2010 and was completed July 2011. Family physician recruitment rate was 79.44% (85/107). Community-based family physicians (n = 79) from 22 medical clinics (39 clusters) completed the study and enrolled a total of 2,395 patients. Figure 1 outlines the CONSORT flow diagram for the study. Tables 1, 2 and 3 outline the baseline characteristics for each treatment group for control and intervention clusters, family physicians and patients, respectively. All analyses were based on the intention to treat model.

**Table 1:** **Baseline characteristics of clusters and physicians randomized to the control and intervention groups**^**1,2,3**^

**Control**																				
**Cluster No.**	**1**	**2**	**3**	**4**	**5**	**6**	**7**	**8**	**9**	**10**	**11**	**12**	**13**	**14**	**15**	**16**	**17**	**18**	**19**	-
**No. of Family Physicians (male/female)**	4 2/2	2 1/1	1 0/1	3 1/2	1 0/1	1 1/0	9 5/4	2 1/1	1 0/1	3 2/1	1 1/0	1 0/1	1 1/0	3 2/1	1 1/0	1 1/0	3 1/2	1 1/0	1 0/1	-
**No. of Patients**	145	31	23	72	33	16	245	67	22	90	34	36	40	67	28	30	100	27	36	-
**Cluster Screening Rate (%)**	34	50	79	74	73	69	55	57	61	59	57	50	70	46	71	77	59	82	51	-
**Membership in Primary Care Quality Improvement Initiative**	Y	N	N	Y	Y	N	Y	N	N	Y	N	N	Y	Y	Y	Y	Y	Y	N	-
**Electronic Medical Record**	Y	N	Y	Y	Y	N	Y	N	N	Y	Y	N	N	Y	Y	N	N	N	N	-
**Intervention**																				
**Cluster No.**	**20**	**21**	**22**	**23**	**24**	**25**	**26**	**27**	**28**	**29**	**30**	**31**	**32**	**33**	**34**	**35**	**36**	**37**	**38**	**39**
**No. of Family Physicians (male/female)**	2 2/0	1 1/0	1 0/1	1 1/0	1 1/0	3 3/0	5 4/1	1 0/1	3 1/2	3 0/3	1 1/0	1 1/0	2 2/0	2 2/0	1 1/1	2 2/0	1 0/1	6 0/6	1 1/0	3 3/0
**No. of Patients**	54	10	32	32	20	96	129	5	92	92	30	32	64	61	20	93	29	178	31	99
**No. of Patients Utilizing the**																				
**Patient Aid**																				
**(Website; Telephone)**	4	-	-	**-**	-	1	1	-	2	4	1	2	1	-	-	1	2	3	-	4
**Cluster Screening Rate (%)**	71	64	66	55	45	62	76	20	64	70	77	79	70	61	80	59	64	63	63	74
**Membership in Primary Care Quality Improvement Initiative**	N	Y	Y	N	N	N	N	N	N	N	Y	Y	N	N	Y	N	Y	Y	Y	Y
**Electronic Medical Record**	Y	Y	N	N	N	Y	Y	N	N	N	N	N	Y	N	N	Y	N	Y	Y	Y

^1^Control treatment group composed of n = 19 clusters, n = 40 family physicians and n = 1,174 patients). Intervention treatment group composed of n = 20 clusters, n = 41 family physicians and n = 1,221 patients.

^2^Average FOBT screening rate = 57% (range: 33.8 to 81.5%) compared to 66.6% (range: 20 to 78.8%) for the intervention group (p < 0.0001). Figures 2 and 3 show the screening rates for the control and intervention group clusters and family physicians, respectively.

^3^Yes = Y, and No = N.

**Table 2:** Baseline characteristics of family physicians randomized to the control and intervention groups

	**Control group (n = 39)**		**Intervention group (n = 40)**	
	**Male (n = 21)**	**Female (n = 18)**	**Male (n = 26)**	**Female (n = 14)**
**Number of family physicians involved in primary care quality improvement initiative(s) (%)**^**1**^				
**Yes**	17 (81.0)	13 (72.2)	8 (30.8)	8 (57.1)
**No**	4 (19.0)	5 (19.0)	18 (27.8)	6 (69.2)
**Number of family physicians in group practice (%)**^**2**^	19 (27.1)	17 (21.5)	25 (31.7)	14 (17.7)
**Number of family physicians with an on-site laboratory (%)**^**3**^	18 (22.8)	18 (22.8)	25 (31.7)	14 (17.7)
**Number of family physicians using electronic medical records (%)**^**4**^	14 (17.7)	11 (13.9)	18 (22.8)	7 (8.9)

^1^Percentage of male and female family physicians in each treatment group involved in a primary care quality improvement initiatives (PIN, UPCON, or PIN + UPCON). 77% of family physicians in the control group were involved in a primary care quality improvement initiative, compared to 40% of family physicians in the intervention group. Table 4 outlines the number of patient in each treatment group according to the primary care quality improvement initiatives.

^2^Four (5.1%) of the collaborating physicians were in solo practice; two males in the control group and one male in the intervention group.

^3^Four (5.1%) of physicians did not have an on-site laboratory; three males in the control group and one male in the intervention group.

^4^50 of 79 family physicians (63.3%) were using electronic medical records.

**Table 3:** Baseline characteristics of patients enrolled in control and intervention treatment groups

	**Control group (n = 1,174)**	**Intervention group (n = 1,221)**
**Gender**		
** Male (%)**	438 (37.3)	468 (38.3)
** Female (%)**	736 (62.7)	753 (61.7)
**Age in years (total)**^**1**^		
**50-54**	119/194 (313)	124/209 (333)
**55-59**	99/164 (263)	123/193 (318)
**60-64**	109/156 (265)	100/163 (268)
**65-69**	65/123 (188)	70/100 (172)
**≥70**	46/89 (135)	51/72 (124)
**Socioeconomic Status (SES)**^**2**^		
**Rural-1**	102 (8.7%)	157 (12.9%)
**Urban-1**	90 (7.7%)	82 (6.7%)
**Urban-2**	324 (27.6%)	316 (25.9%)
**Urban-3**	600 (51.1%)	603 (49.4%)
**Unknown**	58 (4.9%)	63 (5.2%)
**Membership in PIN and/or UPCON**		
**PIN**	419 (35.7%)	314 (25.7%)
**UPCON**	482 (41.1%)	159 (13%)
**None**	274 (23.3%)	747 (61.2%)
**Electronic Medical Records**		
**No**	451 (38.4%)	452 (37.0%)
**Yes**	724 (61.6%)	768 (63.0%)

^1^Males/Females; bracketed values represent totals.

^2^Source: Manitoba Centre for Health Policy, 2010. Based on postal codes. Rural ($22,449 to 148,242); Urban-1 ($14,640 to 42,407); Urban-2 ($42,463 to 68,132); Urban-3 ($68,140 to 406,531). Bracketed values correspond to percentages.

The control group was composed of 19 clusters, 39 community-based family physicians and 1,174 patients. The intervention group was composed of 20 clusters representing 40 community-based family physicians and 1, 221 patients. Family physicians from both groups had similar levels of utilization of electronic medical records (64.1% and 62.5%) and on-site laboratories (92.3% and 97.5%). In addition, the percentage of family physicians in the control and intervention groups who were part of a solo and group clinical practice were similar (92.3% and 97.5%, respectively for group practice). Membership in primary care reform initiatives differed. However, this did not represent a factor in determining differences in FOBT completion rates.

Of the patients participating in the study, approximately 60% were female and 40% male in both groups. Patient age distribution was also similar in the treatment groups.

Table 4 outlines the effect of cluster, physician, and patient level factors on patient FOBT completion rate determined by multi-level logistic regression. Patients in the intervention group had a significantly higher FOBT completion rate (66.6%; n = 805/1209; odds ratio 1.47 with 95% CI 1.06 to 2.03, p < 0.02) compared to those in the control group (56.9%; n = 663/1165). The interclass correlation coefficients were 0.003, 0.009, and 0.217 for the primary group level outcomes of cluster, family physician, and patient.

**Table 4:** Effects of patient level factors on patient fecal occult blood test completion rates

Factor*	**Group**	**FOBT completion**^**1,2**^**(%)**	**Odds ratio (OR)**	**95% Confidence limit**	**p-value**^**3**^
**Treatment**					
	Control	56.9	-	-	-
	Intervention	**66.6**	1.47	1.06, 2.03	0.0001
**Patient Gender**					
	Female	61.6	-	-	-
	Male	62.1	1.07	0.88, 1.29	NS
**Patient Age (years)**					
	50 to 54	51.3	-	-	-
	55 to 59	**60.8**	1.38	1.09, 1.74	0.0001
	60 to 64	**65.1**	1.76	1.38, 2.25	0.0001
	65 to 69	**70.4**	2.33	1.75, 3.10	0.0001
	70 to 74	**72.2**	2.43	1.75, 3.37	0.0001
**Have you ever done an FOBT before?**^**4**^					
	No	43.0	-	-	-
	Yes	**70.6**	3.06	2.51, 3.73	0.0001
**Has a healthcare provider ever suggested you do an FOBT?**^**4**^					
	No	51.5	-	-	-
	Yes	**65.0**	1.54	1.23, 1.92*	0.0001
	Unsure	51.5	1.17	0.80, 1.74	NS

^1^The Intraclass Correlation Coefficients (ICC) for the medical clinic cluster, family physician, and patient level variables were 0.003, 0.009, and 0.217, respectively.

^2^The bolded percentages denote significant differences for each factor.

^3^NS = not statistically significant.

^4^These factors were obtained from the (pre-visit) In-Clinic Patient Survey [18]. There were significant interactions between each factor and cluster (p < 0.0001) as well as family physician (p < 0.0001).

^*^Determined by multi-level logistic regression. FOBT completion rate frequency converted from logit scale for the factor treatment are 0.61for the control and **0.69** for intervention groups (p < 0.02); for the factor patient gender are 0.65 and 0.66 for control and intervention groups, respectively (NS); For patient age are 0.53, **0.60**, **0.66**, **0.73** and **0.72** for the age categories 50 to 54, 55 to 59, 60 to 64, 65 to 69, and 70 to 74, respectively (p < 0.0001); for the survey question “Have you ever done an FOBT before” are 0.48 and **0.73** for the answer ‘no’ and ‘yes’, respectively (p < 0.0001); for the survey question “Has a healthcare provider ever suggested you do an FOBT” are 0.58, **0.68** and 0.62 for the answer ‘no’, ‘yes’ and unsure, respectively (p < 0.0001 for yes and NS for unsure).

Utilization of the patient tools was 13/1,221 (1.07%) for both the website and the nurse-managed telephone support line representing 16 family physicians from 12 clusters. Four males (average age 65 years) and 9 females (average age 61 years) accessed the website and 10/13 (76.9%) completed their FOBT. Four males (average age 70 years) and nine females (average age 57 years) called the telephone support line and 8/13 (61.5%) completed their FOBT. Table 5 outlines the FOBT completion rate by treatment, gender and age.

**Table 5:** Fecal occult blood test completion by treatment, gender, and age category

	**Fecal occult blood test status**^**1,2,3**^									
	**Control group**^**4**^				**Intervention group**^**5**^				**TOTAL**	
	**Male**		**Female**		**Male**		**Female**			
**Age group**	**No**	**Yes (%)**	**No**	**Yes (%)**	**No**	**Yes (%)**	**No**	**Yes (%)**	**No**	**Yes (%)**
**50 to 54**	69	50 (42.0)	107	87 (44.9)	45	79 (63.7)	95	114 (54.6)	316	330 (51.1)
**55 to 59**	43	56 (56.6)	76	88 (53.7)	44	79 (64.2)	64	129 (66.8)	227	352 (60.8)
**60 to 64**	49	60 (55.1)	57	99 (63.5)	27	73 (73.0)	51	112 (68.7)	184	344 (65.2)
**65 to 70**	20	45 (69.2)	39	84 (68.3)	14	56 (80.0)	33	67 (67.0)	106	252 (70.4)
**70+**	14	32 (69.6)	27	62 (69.7)	18	33 (64.7)	13	59 (81.9)	72	186 (72.1)
**TOTAL**	195	243 (55.5)	306	420 (57.9)	148	320 (68.4)	256	481 (65.3)	905	1464 (61.8)

^1^Significant effect of treatment (p < 0.0001) and age (p < 0.0001); no gender or socioeconomic status effects (data not shown).

^2^Values represent number of patients with incomplete FOBT (No) and complete FOBT (Yes) status for males and females in the control and intervention treatment groups.

^3^Bracketed values represent the percentage of patients with complete FOBT status.

^4^Number of missing values due to unknown age or gender was 4 males and 5 females for control group.

^5^Number of missing values due to unknown age or gender was 6 males and 5 females for the intervention group.

**Figure 2:**
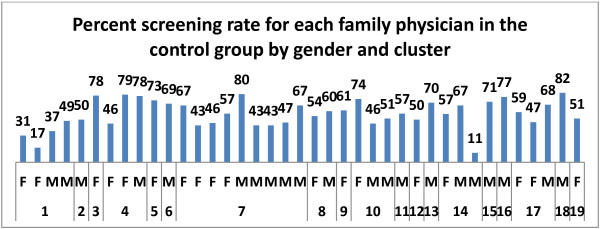
**Screening rate for individual family physicians in the control group by gender and cluster.** There were significant differences in FOBT screening rates among the clusters in the control group (p < 0.0001). Average screening rate for control group is 57%; range 33.8 to 81.5%. F = Female, M = Male. See text for further details.

**Figure 3:**
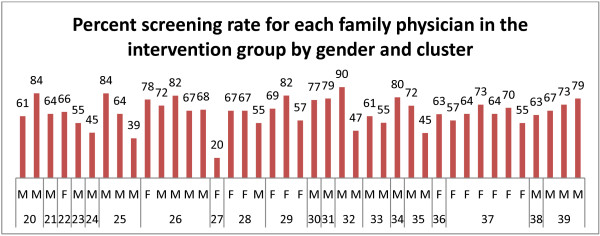
**Screening rate for individual family physicians in the intervention group by gender and cluster.** There were significant differences in FOBT screening rates among the clusters in the intervention group (p < 0.0001). Average screening rate for intervention group is 66.6%; range 20 to 78.8%. F = Female, M = Male. See text for further details.

Although FOBT completion rate was not affected by patient gender or socioeconomic status, there was a significant increase in FOBT completion rate as individuals aged (p < 0.0001). There were also significant differences in FOBT screening rates among clusters within the same treatment group (Figures 2 and 3; p < 0.0001) as well as between treatment groups (p < 0.0001). Similarly, there were significant differences in FOBT screening rates among family physicians within the same cluster and treatment group (p < 0.0001) as well as between treatment groups (p < 0.0001).

Table 6 outlines Patient and Family Physician Follow-Up Survey responses. Fifty-one percent (n = 242/470) of patients whose physician did not suggest the FOBT completed the test. Patients whose physician suggested the FOBT were significantly more likely to complete the test (65%; n = 1140/1755, 95% CI 1.23 to 1.92; p < 0.0001). Compared to patients reporting they had never completed an FOBT in the past (43%; n = 303/705), patients reporting they had were more likely to complete the FOBT (70.6%; n = 1141/1616; 95% CI 2.51 to 3.73; p < 0.0001). Patients in the intervention group reported a significantly higher level (40.2%; 149/371) of discussion about the importance of doing the FOBT compared to the control group (27.6%; 102/369; p < 0.01). However, family physicians in the intervention group (Family Physician Survey) reported that their patients did not ask more questions than usual about CRC, CRC screening, or the FOBT.

**Table 6:** **Patient and family physician reported colorectal cancer screening and fecal occult blood testcommunications**^**1**^

	**Study treatment group**^**2,3**^		
	**Control n = 334 (%)**	**Intervention n = 351 (%)**	**p value**
**Patient Reported Family Physician Communication**			
Did anyone talk to you about CRC during your appointment?	103 **(27.7)**	153 **(40.1)**	0.01
Did anyone tell you why it’s important to do the FOBT?	188 **(50.8)**	228 **(59.7)**	NS
Did you get instructions about how to do the FOBT?	244 **(67.2)**	277 **(73.5)**	NS
Any questions about the test that were not explained?	9 **(2.5)**	8 **(2.1)**	NS
**Family Physician Reported Communication**			
The study protocol caused my patients to ask more questions than usual about:	**Control (n = 33)**	**Intervention (n = 36)**	**p Value**
Colorectal Cancer	16 **(57.1)**	12 **(42.9)**	NS
Colorectal Cancer Screening	18 **(56.3)**	14 **(43.8)**	NS
Fecal occult blood test	19 **(61.3)**	12 **(38.7)**	NS

^1^Survey details can be found in the study protocol [18]; 10 patients from each family physician responded to the Post-Study Patient Survey.

^2^Values represent the number of patients or family physicians responding “Yes” to each question.

^3^Response rate to the patient and physician surveys were 66.7% (685/1142) and 87.3% (69/79 family physicians, respectively).

This study found that patients reporting on the presence of a computer in their home were similar for both the control and intervention groups (90.8%, n = 325/358, of control group patients compared to 87.7%, n = 321/366, in the intervention group) as well as self -reported internet use to answer health related questions.

For those study participants from both the control and intervention groups who completed their FOBT, 25% did so within six days, 50% within 14 days, 75% within 34 days, and 95% within 178 days.

## Discussion and conclusion

A patient aid (refrigerator magnet), promoting a study specific website and nurse-managed telephone line that supplied colorectal cancer screening information, was distributed to patients by their family physician with the goal of increasing colorectal screening rates. The intervention group showed significantly higher fecal occult blood screening rates compared to the control group. This study demonstrates a feasible and effective method of incorporating primary healthcare research into community-based fee-for-service family practice to produce clinically relevant findings.

As a pragmatic trial, this study had very broad inclusion criteria (age and eligibility for CRC screening) representative of the “typical” patient seen by family physicians in daily clinical practice, with little (if any) intervention in the routine physician-patient interaction. Hence, the findings should be generalizable to community-based family practice and, therefore provide meaningful and relevant implications for clinical family practice. Earlier self-reported estimates of CRC screening rates in Manitoba were between 38 to 42% [7,9] with a more recent self-reported provincial screening rate of 64% [8]. However, both surveys combined FOBT, colonoscopy and flexible sigmoidoscopy in the definition of colorectal cancer screening. Hence, we are unable to compare the screening rates found in the study to those in the surveys to determine whether the control group rates are indicative of ‘typical’ rates or whether this group experienced an intervention effect along with the intervention group. Patients in the intervention group did, however, have a significantly higher FOBT completion rate than those in the control group. This may be due to physicians being reminded to have a conversation about the importance of CRC screening with their patients through their role in handing out the study intervention magnet, despite the self-report that this was not the case. Previous research supports the apparent lack of veracity of physician responses to questions of this nature [24,25]. The striking findings of this study are the very low rates of utilization of the information resources provided. Neither internet nor telephone access were an issue for the intervention group. Instead, direct communication from the family physician underscoring the importance of screening seems to have obviated the need for access to the educational materials. The mere provision of the patient aid, by a family physician to their patient, may have underscored importance of, and the physician’s recommendation to do the test. If this is the case, it is clear evidence of the value of family physicians in promoting CRC screening. It also provides a simple and effective method for family physicians to improve patient FOBT compliance without placing demands on the time constraints a clinician has to address all other health issues during the physical exam. A future question for investigation based on the study findings is whether physicians must provide a patient aid to their patients (such as the magnet in this study), or whether it is possible to use an even simpler strategy?

Findings from the multi-level regression analysis demonstrate that the most significant factors affecting CRC screening rate are family physician-patient communication and patient level factors. The Post-Study Patient Follow-Up Survey showed that a significantly higher number of patients in the intervention group (40.0% versus 27.7%) reported that someone had talked to them about CRC during their clinic visit. A systematic review conducted in 2010 demonstrated that there is high quality evidence supporting one-on-one interactions in increasing absolute CRC screening rates (between 15 to 42 percent) and provided strong evidence to support the effectiveness of patient reminders in increasing absolute CRC screening rates (5 to 15%) [26]. It is possible that for some patients in the intervention group of this study, the magnet functioned as both a personal recommendation from their health care provider and a reminder to undergo CRC screening. An important finding of this study is that a much more practical and clinically relevant method may exist for the one-on-one interaction required by a family physician to convey the importance of CRC screening to their patient’s. In fact, our findings suggest a very brief show of support of the FOBT is necessary compared to the increased time reported in the literature necessary to increase CRC screening rates [10].

The implications of our findings for clinical practice suggest family physicians specifically focus their efforts to support CRC screening behavior on men and women between the ages 50 to 60 years, which represent the group with the lowest screening rates and the most benefit to gain from screening. If supporting uptake of FOBT screening is successful for this age group of men and women, our findings show that these individuals are much more likely to engage in repeat screening. Hence, the possible outcome is reduction in incidence and mortality from CRC in the age group most highly burdened by the disease (60 to 69 years of age) [7]. Our results confirm those reported in that FOBT uptake tends to increase with age among men and women [27].

There are a few limitations of the study. Inclusion of the In-Clinic Patient Survey in both study groups may have raised patient awareness about CRC and screening sufficiently to affect/improve screening rates in the control group. In addition, those family physicians agreeing to participate in the study may have done so with awareness that their patients have a relatively higher screening rate. The results may be limited to fee-for-service family physicians and those in urban areas. Some providers were involved in quality improvement interventions (PIN and UPCON) [18,28]. These clinics may have a specific approach to screening that has already lead to improvements in patient CRC screening rates and functioning/practice may be more evidence-based compared to those that are not involved in these initiatives. In these groups, the study intervention may have minimal effect. There was uneven distribution of physicians with no membership in PIN or UPCON in the control (n = 9) and intervention (n = 24) groups. However, the multi-level regression analysis failed to demonstrate a relationship between participation in either initiative and treatment effect on FOBT status.

The study design is unique in that intraclass correlation coefficients (ICC) values for colorectal cancer screening rates are provided for three levels of clustering: at three levels; the patient, family physician and medical clinic. To our knowledge, this is the first paper to present ICCs for CRC screening rates at the primary care practice level. This is a beneficial contribution to the scientific literature by supporting future appropriate design and analysis of primary prevention trials. For FOBT completion rate, the ICCs were small (0.003 and 0.009 for medical clinics and family physicians, respectively) and the design effects (1.12 and 1.02 for medical clinic and family physician, respectively) would result in a power calculation very similar to that of a simple randomized trial. The low ICCs demonstrate the minimal extent to which patient FOBT screening rate was influenced by clustering of observations in higher level groups (family physician and medical clinic/cluster). The findings demonstrate that FOBT screening status is influenced most strongly by factors at the level of the patient, whereas the family physician and clinic (cluster) and factors have less of an impact on FOBT screening rates.

As the number of primary care prevention trials are increasing, future study protocols will increasing rely on cluster randomized designs. Reporting of intraclass correlation coefficients is necessary to accurately calculate sample size at the level of community-based primary care practice. This paper adds to the literature by providing intraclass correlation coefficients for colorectal cancer screening rates at the primary care practice level (medical clinic, family physician, and patient) that will assist in future design and analysis of appropriate clustered primary prevention trials.

Our results provide family physicians a clinically relevant practice tool that is responsive to the requirements of daily clinical practice and both feasible and effective in increasing colorectal cancer screening rates among their patients.

## Abbreviations

CRC: Colorectal cancer; FOBT: Fecal occult blood test; FP: Family physician; ICC: Intraclass correlation coefficient; PIN: Physician integrated network; UPCON: Uniting primary care and oncology/urban primary care oncology network.

## Competing interests

I/We declare the authors have no competing interests. All authors declare no financial relationships with any organizations that might have an interest in the submitted work and no other relationships or activities that have influenced the submitted work. Competing interests: none declared.

## Authors’ contributions

KC and AK provided substantive intellectual contributions to the study conceptualization, design, organization and data analysis and interpretation. KC wrote the first draft of this manuscript providing substantive intellectual content and was responsible for family physician recruitment and retention, study implementation and data acquisition. KC, AK, and PM provided substantive intellectual contributions to the statistical design of the study. GC, KC, AK contributed substantially to the statistical analysis of the data. All authors contributed to the interpretation of the study findings. AK, PM, JS, DT, ML, SM, and GC were involved in critically reviewing the manuscript for important intellectual content. All authors had full access to and can take responsibility for the data and analyses. The CIHR/CCMB Primary Care Oncology-New Emerging Team provided their intellectual contributions to the study design and interpretation of the study findings at quarterly workshops. All authors gave final approval of the manuscript version submitted for publication. Dr. Cornelius Woelk is the designated CIHR/CCMB PCO-NET group representative.

## Pre-publication history

The pre-publication history for this paper can be accessed here:

http://www.biomedcentral.com/1471-2407/14/263/prepub

## CONSORT and extension statements

This pragmatic, cluster randomized controlled trial was performed and the manuscript written according to the CONSORT guidelines, including both cluster and pragmatic extension guidelines.

## Data sharing

No additional data available.
